# Human Pancreatic Cancer Organoids

**DOI:** 10.1111/cpr.70210

**Published:** 2026-04-23

**Authors:** Aina He, Xiaolin Lin, Xiaozhe Qian, Jiasheng Mu, Zhonghua Tao, Jin Gu, Yonggang Wang, Ting Han, Hongling Zhao, Yalong Wang, Jian Zhang, Zunqiang Zhou, Ligang Xing, Changchun Zhou, Dongyuan Zhu, Zengjun Liu, Gang Chen, Qinghe Zhou, Xiaoguang Wang, Shasha Zhao, Xiao Liu, Dongyan Cao, Bin Yu, Zebing Liu, Jiabei Wang, Gengming Niu, Guiying Wei, Maorong Chen, Weiping Wang, Xiaonan Kang, Junmei Zhou, Shuai Gong, Ying Xie, Jianming Zhang, Xianming Kong, Chunyan Dong, Yanmei Zou, Lina Tang, Aijin Ma, Hua Xiong, Xuemei Liu, Tongbiao Zhao, Jie Hao, Xiuying Xiao, Dongxi Xiang

**Affiliations:** ^1^ Department of Oncology Shanghai Jiao Tong University Affiliated Sixth People's Hospital Shanghai China; ^2^ Department of Oncology Renji Hospital Affiliated to Shanghai Jiao Tong University School of Medicine Shanghai China; ^3^ Department of Thoracic Surgery Renji Hospital, School of Medicine, Shanghai Jiao Tong University Shanghai China; ^4^ Department of General Surgery Xinhua Hospital, Affiliated to Shanghai Jiao Tong University, School of Medicine Shanghai China; ^5^ Department of Medical Oncology Fudan University Shanghai Cancer Center, Department of Oncology, Shanghai Medical College, State Key Laboratory of Genetic Engineering, School of Life Sciences, Human Phenome Institute, Fudan University Shanghai China; ^6^ Department of Hepatobiliary and Pancreatic Surgery Affiliated Hospital of Zunyi Medical University Zunyi China; ^7^ Beijing Institute for Stem Cell and Regeneration Medicine Beijing China; ^8^ Standard Committee, Chinese Society for Cell Biology Shanghai China; ^9^ The MOE Basic Research and Innovation Center for the Targeted Therapeutics of Solid Tumors, School of Basic Medical Sciences, Institute of Biomedical Innovation, Jiangxi Medical College, Nanchang University Nanchang China; ^10^ Department of Medical Oncology Fudan University Shanghai Cancer Center Shanghai China; ^11^ Department of Surgery Shanghai Jiao Tong University Affiliated Sixth People's Hospital Shanghai China; ^12^ Department of Radiation Oncology Shandong Cancer Hospital and Institute, Shandong First Medical University, Shandong Academy of Medical Science Jinan China; ^13^ Biobank, Cancer Research Center, Shandong Cancer Hospital and Institute, Shandong First Medical University, Shandong Academy of Medical Sciences Jinan China; ^14^ Rare Tumors Department Shandong Cancer Hospital and Institute, Shandong First Medical University and Shandong Academy of Medical Sciences Jinan China; ^15^ Jiaxing Key Laboratory of Basic Research and Clinical Translation on Orthopedic Biomaterials, Department of Orthopaedics, The Second Affiliated Hospital of Jiaxing University Jiaxing China; ^16^ Department of Hepatobiliary Surgery Affiliated Hospital of Jiaxing University Jiaxing China; ^17^ State Key Laboratory of Systems Medicine for Cancer, Shanghai Cancer Institute, Shanghai Key Laboratory of Cancer System Regulation and Clinical Translation, Jiading Branch, Renji Hospital, Shanghai Jiao Tong University School of Medicine Shanghai China; ^18^ Department of Pathology Renji Hospital, School of Medicine, Shanghai Jiao Tong University Shanghai China; ^19^ Department of Hepatobiliary Surgery The First Affiliated Hospital of USTC, Division of Life Sciences and Medicine, University of Science and Technology of China Anhui China; ^20^ Shanghai OneTar Biomedicine Shanghai China; ^21^ Center for Life Science, Yunnan Key Laboratory of Cell Metabolism and Diseases, School of Life Sciences Yunnan University Kunming China; ^22^ Department of Pharmacology and Pharmacy Li Ka Shing Faculty of Medicine, The University of Hong Kong Hong Kong China; ^23^ Department of Biobank Renji Hospital Affiliated to Shanghai Jiao Tong University School of Medicine Shanghai China; ^24^ Department of Central Laboratory Shanghai Children's Hospital, School of Medicine, Shanghai Jiao Tong University Shanghai China; ^25^ Division of Gastroenterology and Hepatology Renji Hospital Affiliated to Shanghai Jiao Tong University School of Medicine Shanghai China; ^26^ Life Sciences Institute, Guangxi Medical University Nanning China; ^27^ Institute of Translational Medicine, Zhangjiang Institute for Advanced Study, Shanghai Jiao Tong University Shanghai China; ^28^ Collaborative Research Center for Biomedicines, Shanghai University of Medicine and Health Sciences Shanghai China; ^29^ Department of Oncology East Hospital Affiliated to Tongji University, Tongji University School of Medicine Shanghai China; ^30^ Department of Oncology Tongji Hospital, Tongji Medical College, Huazhong University of Science and Technology Wuhan China; ^31^ Beijing Technology and Business University Beijing China; ^32^ Department of Gastroenterology Digestive Disease Hospital, Affiliated Hospital of Zunyi Medical University Zunyi China; ^33^ Key Laboratory of Organ Regeneration and Reconstruction, State Key Laboratory of Stem Cell and Reproductive Biology, Institute of Zoology, Chinese Academy of Sciences Beijing China; ^34^ National Stem Cell Resource Center, Institute of Zoology, Chinese Academy of Sciences Beijing China; ^35^ Key Laboratory of Early Prevention and Treatment for Regional High Frequency Tumor (Guangxi Medical University), Ministry of Education Nanning China; ^36^ Jiaxing Organoid Center Jiaxing Zhejiang China

## Abstract

This guideline establishes a comprehensive framework for the application of patient‐derived pancreatic cancer organoids. It outlines the stringent technical requirements and testing methods necessary to ensure high fidelity to the original tumor tissue, including morphological assessment, pathological biomarker expression (e.g., CK19, CK7), and genetic concordance (e.g., NRG1, KRAS). By standardizing protocols for in vitro culture, microbiological safety, and STR authentication, this consensus aims to ensure the reproducibility, safety, and stability of organoid models, thereby accelerating their integration into basic research, drug discovery, and precision medicine.

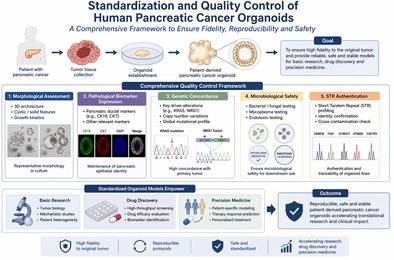

The clinical treatment of pancreatic cancer patients faces many challenges [1]. Traditional chemotherapies and targeted therapies show limited efficacy, and immunotherapies also bring no significant benefits. Accordingly, the clinical demand for precision medicine from pancreatic cancer patients is very urgent. Over the past decade, researchers utilizing organoids to mimic patient tumours have experienced tremendous progress [2]. Patient‐derived organoids are able to recapitulate the physiological, pathological, and genetic characteristics of their original tumour tissues in vitro, thus offering unprecedented opportunities for drug development and precision medicine [3]. The “Human Pancreatic Cancer Organoids” is part of a series of guidelines for human cancer organoids in China, jointly drafted by experts from the Chinese Society for Cell Biology and its branches [4], and its Chinese version was initially released on October 28th, 2024. This standard document outlines terminologies, technical requirements and assessment protocols for human pancreatic cancer organoids, and applies to their production, evaluation procedures and quality control. Publication of the English version for this standard document aims to assist relevant institutions in endorsing, establishing, and implementing best practices, thereby advancing the international standardization of human pancreatic cancer organoids in both fundamental research and clinical applications.

## 1 Scope

This document specifies the general requirements, technical requirements, testing methods, inspection rules, instructions for use, labelling, transportation, and storage for human pancreatic cancer organoids.

## 2 Normative References

The contents of the following documents constitute indispensable provisions of this document by means of normative references in the text. For dated references, only the edition cited applies. For undated references, the latest edition of the referenced document (including all amendments) applies.

*Pharmacopoeia of the People's Republic of China* (2025 Edition, Part III).

## 3 Terms and Definitions

The following terms and definitions apply to this document.

### 3.1 Organoids

Three‐dimensional (3D) cultures that grow from stem cells or progenitor cells in vitro, are capable of self‐organization and renewal, consist of organ‐specific cell types and can mimic the in vivo structure and specific function of the original tissue [5].

### 3.2 Human Pancreatic Cancer Organoids

Organoids that are developed from pancreatic tumour cells of patients pathologically diagnosed with pancreatic cancer, and can simulate the characteristics of original pancreatic cancer tissues.

### 3.3 Organoid Passage

Process of dissociating existing organoids into smaller fragments, or single cells via physical, chemical, or biological methods, then inoculating and growing them in vitro under the same conditions as the original culture.

### 3.4 Organoid Cryopreservation

Freezing process by which organoids are temporarily preserved under low temperature in an inactive state for maintaining their cellular compositions, gene expression profiles, and functional properties.

### 3.5 Organoid Thawing

Process by which frozen organoids regain growth and metabolic vitality from an inactively state.

## 4 Abbreviations

The following abbreviations apply to this document.

**Table:** 

3D: three dimension	H&E: haematoxylin and eosin
DAB: diaminobenzidine	PBS: phosphate‐buffered saline
DMSO: dimethyl sulfoxide	PCR: polymerase chain reaction
DNA: deoxyribonucleic acid	STR: short tandem repeat

## 5 General Requirements

### 5.1 Raw Materials

5.1.1 The acquisition of raw materials shall comply with domestically recognized ethical standards and local laws [6, 7].
5.1.2 Depending on the intended use, donor evaluation criteria shall be established for the research and production of human pancreatic cancer organoids.

### 5.2 Process and Information Management

5.2.1 Critical factors that may influence product quality during the procurement, preparation, testing, transportation, and storage shall be documented, and a unique identity shall be implemented to ensure traceability throughout the process.
5.2.2 The minimum retention period for records shall be clearly defined to ensure the integrity and security of documentation.

## 6 Technical Requirements

### 6.1 Morphology

Human pancreatic cancer organoids exhibit various morphological features including dense‐cystic, spherical, or round‐tubular shapes under optical microscopy.

### 6.2 Pathological Features

6.2.1 Cells in the organoids should maintain the atypical characteristics of tumour cells from the original tumour tissue, such as hyperchromatic nuclei, abnormal mitotic figures, and disrupted nuclear‐cytoplasmic ratio, etc.
6.2.2 Immunohistochemical testing for relevant biomarkers shall be performed on the organoids, and the results shall be generally consistent with the original tumour tissues. Markers tested include, but are not limited to, CK19 and CK7.

### 6.3 Genetic Characteristics

Genetic variation testing should be performed on the organoids, and the results should be essentially the same as the genetic variation results of the original tumour tissue. Genes tested include, but are not limited to *NRG1* and *KRAS*.

### 6.4 In Vitro Culture and Growth

6.4.1 Human pancreatic cancer organoids derived from pancreatic cancer patient tissues or cells shall be capable of being passaged for at least three generations after the initial culture in vitro.
6.4.2 Post‐passage organoids shall be reconstructed in vitro into new analogous organoids, and their morphology, pathological characteristics and genetic characteristics should be consistent with those of the pre‐passage organoids.

### 6.5 Survival Rate of Organoids

The survival quantity of thawed human pancreatic cancer organoids from cryopreservation should not be less than 50% of the number before freezing. And the surviving organoids shall be able to be passaged in vitro.

### 6.6 Microorganisms

Organoids shall be negative for the testing of fungi, bacteria, mycoplasma, and virus.

### 6.7 STR

The identity of organoids shall match that of the donor tissue by STR analysis.

## 7 Testing Methods

### 7.1 Morphology

Organoids are cultured three dimensionally in vitro, and observed using optical microscopy.

### 7.2 Pathological Features

Exemplary test methods can be found in Supporting Information Annex A.

### 7.3 Genetic Characteristics

Exemplary test methods can be found in Supporting Information Annex B.

### 7.4 In Vitro Culture and Growth

Organoids cultured three dimensionally in vitro can be photographed using optical microscopy, with a scale bar for measuring their diameters and performing quantitative analysis.

### 7.5 Survival Rate of Organoids

Exemplary test methods can be found in Supporting Information Annex C.

### 7.6 Microorganisms

#### 7.6.1 Fungi and Bacteria

The “1101 Sterility Inspection Method” in *Pharmacopoeia of the People's Republic of China* (2020 Edition, Part III) should be followed.

#### 7.6.2 Mycoplasma

The “3301 Mycoplasma Inspection Method” in *Pharmacopoeia of the People's Republic of China* (2020 Edition, Part III) should be followed.

#### 7.6.3 Exogenous Viral Factors

The “3302 Exogenous Viral Factors Inspection Method” in *Pharmacopoeia of the People's Republic of China* (2020 Edition, Part III) should be followed.

### 7.7 STR

Exemplary test methods can be found in Supporting Information Annex D.

## 8 Instructions for Use

The instructions should include at least the following information:

**Table:** 

(a) Organoid code	(h) Transportation conditions
(b) Passage number	(i) Contact information
(c) Organoid quantity	(j) Usage instructions
(d) Production date	(k) Execution standard number
(e) Batch number	(l) Production address
(f) Manufacturing organization	(m) Postal code
(g) Storage conditions	(n) Precautions

*Note:* Endotoxin results should be provided upon user request.

## 9 Labelling

The labels should include at least the following information:

**Table:** 

(a) Organoid code	(d) Batch number
(b) Passage number	(e) Manufacturing organization
(c) Organoid quantity	(f) Production date

## 10 Transportation and Storage

### 10.1 Transportation

10.1.1 The mode and conditions of transportation should be selected according to the requirements for the use of human pancreatic cancer organoids to ensure their biological properties, safety, stability, and efficacy.
10.1.2 The transportation of human pancreatic cancer organoids should consider, but not be limited to, factors such as the characteristics of the organoids, the container carrying the organoids, the transportation route, transportation conditions, transportation equipment, transportation modes, potential risks, and necessary safeguards.
10.1.3 The control measures for transportation conditions shall include, but not be limited to, temperature range, vibration control, contamination prevention, equipment performance, and appropriate packaging.
10.1.4 Relevant inspection and technical guidance documents should be provided upon user request.
10.1.5 The package shall be checked during transportation, and if necessary, additional freezing sources (e.g., dry ice and liquid nitrogen) should be added to maintain the appropriate transportation temperature.

### 10.2 Storage [8, 9]

10.2.1 Optimized cryopreservation protocols and methods shall be employed to minimize the damage to human pancreatic cancer organoids during cryopreservation and thawing processes, ensuring their normal functionality is minimally affected.
10.2.2 The cryopreservation information for human pancreatic cancer organoids shall be documented, including but not limited to:

**Table:** 

(a) Organoid code	(e) Freezing date
(b) Batch number	(f) Cryoprotectant composition
(c) Organoid quantity	(g) Name of the operator.
(d) Passage number

10.2.3 The storage conditions for human pancreatic cancer organoids should be documented, including but not limited to:
  - Storage conditions;
  - Storage date;
  - Storage duration;
  - Name of the operator.

## Author Contributions

Aina He, Xiaolin Lin, Xiaozhe Qian, Jiasheng Mu, Zhonghua Tao, Jin Gu, Yonggang Wang, Ting Han, Hongling Zhao, Yalong Wang, Jian Zhang, Zunqiang Zhou, Ligang Xing, Changchun Zhou, Dongyuan Zhu, Zengjun Liu, Gang Chen, Qinghe Zhou, Xiaoguang Wang, Shasha Zhao, Xiao Liu, Dongyan Cao, Bin Yu, Zebing Liu, Jiabei Wang, Gengming Niu, Guiying Wei, Maorong Chen, Weiping Wang, Xiaonan Kang, Junmei Zhou, Shuai Gong, Ying Xie, Jianming Zhang, Xianming Kong, Chunyan Dong, Yanmei Zou, Lina Tang, Aijin Ma, Hua Xiong, Xuemei Liu, Tongbiao Zhao, Jie Hao, Xiuying Xiao, Dongxi Xiang critically read and revised the manuscript.

## Funding

This work was supported by National Natural Science Foundation of China, 82573430, 82173358, 62202304, 82403651; Shanghai Municipal Health Commission, 2025ZZ1023, 2024ZZ2032, 2025ZZ1032; Science and Technology Commission of Shanghai Municipality, 23141901000.

## Ethics Statement

The authors have nothing to report.

## Consent

The authors have nothing to report.

## Supporting information


**Data S1:** cpr70210‐sup‐0001‐Supinfo.docx. **Annex A (Informative):** Organoid histopathology testing (Paraffin Embedding Method).
**Annex B (Informative):** Organoid Gene Mutation Testing.
**Annex C (Informative):** Quantification of Organoid Survival Rates (Calcein‐AM Staining Method).
**Annex D (Informative):** Organoid Authentication by STR Profiling.

## Data Availability

The data that support the findings of this study are available on request from the corresponding author. The data are not publicly available due to privacy or ethical restrictions.
