# Supplementary material for: Human Pancreatic Cancer Organoids
Source: Cell Prolif. 2026 Apr 23;59(10):e70210. doi: 10.1111/cpr.70210 (PMC13326021; doi:10.1111/cpr.70210)
Supplement: Supplementary file 1 [file CPR-59-e70210-s001.docx]

**Supplementary information**

**Annex A**

**(Informative)**

**Organoid histopathology testing (Paraffin Embedding Method)**

**A.1 Instruments**

A.1.1 Paraffin-embedding Machine

A.1.2 Paraffin-slicer

A.1.3 Optical Microscope

**A.2 Reagents**

Unless otherwise specified, the reagents used should be analytically pure, and the water used for testing should be deionized water.

A.2.1 Paraffin section preparation reagents: prepare reagents required for paraffin embedding according to the corresponding requirements, including fixing solution, dehydration solution, paraffin, dewaxing solution, rehydration solution, ethanol, xylene, and neutral resin.

A.2.2 H&E staining reagent: hematoxylin, eosin.

**A.3 Testing Protocol**

A.3.1 Sample Preparation and Fixation

The organoids cultured *in vitro* and matrigel are pipetted out then transferred into a 15 mL centrifuge tube. Collect the organoids by centrifugation and discard the supernatant. Fix the organoids with 4% paraformaldehyde for 15-30 minutes.

A.3.2 Preparation of Paraffin sections for Organoids

The organoid samples are fixed, dehydrated, hyalinized, and immersed in paraffin with a paraffin-embedding machine according to the paraffin-section method. Cut the embedded organoid paraffin blocks into slices with standard thickness using a paraffin slicer.

A.3.3 H&E Staining

Paraffin sections of organoids are dewaxed, rehydrated, stained with hematoxylin and eosin, then dehydrated with ethanol, hyalinized by xylene, and sealed with neutral resin.

A.3.4 Immunohistochemistry Staining

Paraffin sections of organoids are dewaxed, rehydrated, antigen repaired and blocked with blocking solution, sections are then incubated with the primary antibodies and washed with PBS, followed by incubating with the second antibodies and washed with PBS. Perform the color reaction with DAB and add water to terminate. The sections are dyed by hematoxylin, hyalinized with hydrochloric acid and flushed, then dehydrated with ethanol, hyalinized by xylene, and sealed by neutral resin.

**A.4 Result Analysis**

The testing results obtained are analyzed and interpreted by personnel qualified for pathological diagnosis, and these results of organoids should be consistent with the results of the original tumor tissue.

**Annex B**

**(Informative)**

**Organoid Gene Mutation Testing**

**B.1 Instruments**

Centrifuge.

**B.2 Reagents**

Cell DNA extraction kit.

**B.3 Sample Storage**

The samples are prepared and stored under -80℃ or below.

**B.4 Testing Protocol**

B.4.1 Sample Preparation

Cultivate the organoids *in vitro* in matrigel until they reach a stable growth state, pipette the matrigel up and down to break it and transfer the mixture in a centrifuge tube, then the organoids are collected by centrifugation, and the supernatant is discarded.

B.4.2 DNA Extraction

Perform genomic DNA extraction from organoids and the corresponding tumor tissues according to the manufacture instructions of the cell DNA extraction kit.

B.4.3 DNA Sequencing

DNA from organoid samples and the corresponding tumor tissues were sent to institutions qualified for clinical genetic testing for 1st or 2nd generation sequencing testing, or the genetic loci are tested by amplification refractory mutation system (ARMS) method or droplet digital PCR (ddPCR) method.

**B.5 Result Analysis**

Analyze the mutant loci and compare the sequencing results concordance between organoid and the corresponding tumor tissues.

**Annex C**

**(Informative)**

**Quantification of Organoid Survival Rates (Calcein-AM Staining Method)**

**C.1 Instruments**

C.1.1 Inverted Microscope

C.1.2 Fluorescence Microscope

**C.2 Reagents**

Unless otherwise specified, the reagents used should be analytically pure, and the water used for testing should be deionized water.

C.2.1 Dimethyl sulfoxide (DMSO) for cell culture.

C.2.2 PBS (pH 7.4).

C.2.3 Preparation of the storage solution (Calcein-AM solution): Prepare the 2 mmol/L Calcein-AM solution with DMSO.

**C.3 Testing Protocol**

C.3.1 Quantification of Total Organoids

Place the organoids under the inverted microscope to observe their morphology and status. Determine whether the organoid morphology meets the requirements of 6.1 by visual observation, and count the number of organoids with a diameter ≥ 20 μm.

C.3.2 Quantification of Surviving Organoids

Add the Calcein-AM storage solution into the organoid culture medium until the final concentration reaching 0.2 μmol/L, and incubate the mixture for 60 minutes at 37℃. Then wash the medium gently with PBS and add fresh medium. The organoids are observed and photographed by fluorescence microscope at 490 nm excitation wavelength and 515 nm emission wavelength. Living organoids appear green with clear edges. Count the number of living organoids with a diameter ≥ 20 μm.

**C.4 Calculation of Organoid Survival Rates**

The organoid survival rates are calculated according to the formula (C.1):

X = (N_Living_ /N_Total_) × 100% ...... (C.1)

In this formula:

X: The organoid survival rate;

N_Living_: The number of living organoids;

N_Total_: The number of total organoids.

**C.5 Result Analysis**

Repeat the step C.3 twice more, then calculate the average of the three results, and record it as the average survival rate of organoids.

**C.6 Accuracy**

The absolute difference between the results of three independent determinations obtained under conditions of repeatability should not exceed 10% of the arithmetic mean.

**Annex D**

**(Informative)**

**Organoid Authentication by STR Profiling**

**D.1 Instruments**

D.1.1 Centrifuge

D.1.2 PCR-Cycler

D.1.3 Capillary Electrophoresis System

D.1.4 Micro-Ultraviolet Spectrophotometer

**D.2 Reagents**

D.2.1 Cell DNA Extraction Kit

D.2.2 STR DNA Profiling Kit

**D.3 Sample Storage**

The samples are prepared and stored under -80℃ or below.

**D.4 Testing Protocol**

D.4.1 Sample Preparation

Cultivate the organoids *in vitro* in matrigel until they reach a stable growth state, pipette the matrigel up and down to break it and transfer the mixture in a centrifuge tube, then the organoids are collected by centrifugation, and the supernatant is discarded.

D.4.2 DNA Extraction

a) Perform genomic DNA extraction from organoids and the corresponding tumor tissues according to the manufacture instructions of the cell DNA extraction kit.
b) Measure the absorbance of extracted DNA by micro-Ultraviolet spectrophotometer to ensure that the ratio of A260/A280 is between 1.8 and 2.0.
c) Sample requirements: DNA volume ≥ 20 μL, concentration ≥ 50 ng/μL.

D.4.3 PCR Amplification

a) Perform STR loci according to the standard DNA PCR amplification methods or the commercially approved kit instructions.
b) Set the negative control group, the sample detection group and the positive control group. Use sterile water as template for PCR amplification in the negative control group, then use DNA extracted from organoids and their corresponding tumor tissues as templates for the sample detection group, and the positive control group are subjected to amplification using a given DNA template.
c) Detect the PCR products of three groups by agarose gel electrophoresis. Clear target band should be observed in the positive control group but not in the negative control group.

D.4.4 STR Genotyping

Detect PCR products by capillary electrophoresis system to obtain STR profile data. The PCR banding pattern of organoids and their corresponding tumor tissues should be consistent.

**D.5 Result Analysis**

D.5.1 When STR alleles contain the same number of repeats, only one allele peak shall appear in the profile. When they contain different numbers of repeats, two allele peaks shall appear in the profile. The test is considered valid when no allele peaks appear in the negative control group and the positive control group result is consistent with its standard genotyping data.

D.5.2 Under the premise of valid detection, if more than two allelic peaks are present at the STR locus of the tested sample, the sample shall be determined to be cross-contaminated after repeated experiments to exclude interferences such as mutations in the primer binding region.
